# Fracture load and three‐dimensional internal void analysis of direct composites used to restore worn molars

**DOI:** 10.1111/eos.70104

**Published:** 2026-05-06

**Authors:** Topias Yli‐Urpo, Jorma Määttä, Auli Suominen, Pekka K. Vallittu, Timo Närhi, Anna‐Maria Le Bell‐Rönnlöf, Jasmina Bijelic‐Donova

**Affiliations:** ^1^ Institute of Dentistry University of Turku Turku Finland; ^2^ Institute of Biomedicine, Faculty of Medicine University of Turku Turku Finland; ^3^ Department of Community Dentistry, Institute of Dentistry University of Turku Turku Finland; ^4^ Department of Biomaterials Science and Turku Clinical Biomaterials Centre Institute of Dentistry University of Turku Turku Finland; ^5^ Oral Health Services Wellbeing Services County of Southwest Finland Turku Finland; ^6^ Department of Prosthetic Dentistry and Stomatognathic Physiology, Institute of Dentistry University of Turku Turku Finland

**Keywords:** air bubble analysis, occlusal veneer, particulate‐filled composite, short‐fiber‐reinforced composite, water storage, worn teeth

## Abstract

This study evaluated (1) the load‐bearing capacity of molars restored with direct occlusal veneers fabricated either from short‐fiber‐reinforced composite (SFRC) or particulate‐filled composite (PFC) and the presence of internal voids within the materials, and (2) effect of water storage on the fracture load and correlation between internal voids and fracture load. Sixty human molars (*n* = 10/group) were prepared with flat occlusal surfaces and preserved enamel margins simulating worn teeth. Conventional molds were taken for each tooth to control the preparation depth and transparent molds to restore the teeth to their original shape. Following bonding procedure, teeth were restored with flowable SFRC, flowable PFC, or condensable PFC using either injectable or pressing technique. Restored teeth were stored in water for 1 d or 6 months before the static loading test. Fracture mode and three‐dimensional internal void analyses were additionally conducted. SFRC occlusal veneers showed significantly higher fracture load (3661 ± 446 N) than both PFC materials, flowable PFC (2064 ± 372 N) and condensable PFC (2183 ± 563 N). SFRC redirected the fractures towards the periphery of the restoration. Prolonged water storage duration or single voids did not adversely affect the fracture load. The use of SFRC as occlusal veneers for restoring molars improves the fracture load and the fracture propagation.

## INTRODUCTION

Tooth wear is a multifactorial condition with increasing global prevalence, currently affecting approximately 40% of the population [1, 2]. Its etiology includes attrition, erosion, abrasion, and abfraction [1]. Tooth wear can cause sensitivity, increase caries risk, and cause temporomandibular disorders, making its management an important aspect of restorative dentistry [2]. Prevention is the primary management strategy. However, when patients’ concerns or symptoms arise, restorative treatment might be indicated [3]. Traditional restorative treatment options, such as full coverage indirect restorations, are important strategies for managing tooth wear. Nevertheless, they involve removal of significant amount of sound tooth structure, and instead less invasive and more cost‐effective alternatives should be considered [3]. The development of material formulations and adhesive techniques has enabled the possibility to select between direct or indirect resin composite and ceramic restorations for restoring worn teeth [4, 5]. Indeed, contemporary resin composites have clinically demonstrated promising performance in restoring occlusal surfaces of posterior teeth [6, 7], but direct restorations for the management of worn teeth are usually extensive and complex. Consequently, they need more time to be made and may need maintenance follow‐up [5]. Indirect restorations provide good approximal contacts and occlusal morphology, but their disadvantage is the need for temporalization. In order to avoid provisional restorations and to utilize the effect of sealing the dentin immediately, in the borderline cases, usually the direct technique is the choice of treatment [4, 8]. The choice is supported by the evolving evidence that contemporary resin composites used to restore severe tooth loss or worn teeth have fairly improved mechanical properties than their precursors [9, 10, 11] and experimentally perform satisfactorily when used to increase vertical dimension of occlusion even when applied as thin direct resin composite occlusal veneers [12]. These restorations are usually designed in a wax‐up or a digital mock‐up and transferred to the teeth using various transparent index molds, either by injecting or pressing the resin composite material over the tooth surface. This procedure allows precise replication of the designed shape, and results are controlled and esthetically pleasing. The direct treatment is reversible and allows future changes or upgrades (e.g., switching to ceramic restorations). Hence, it could be concluded that direct restorations are a viable option for restoring worn teeth [6, 13].

Extensive posterior direct composite restorations, however, fail mainly due to restoration or tooth fractures, and secondary caries [6]. In addition, chipping of the resin composite [14], marginal discrepancy, and discoloration [15] are common findings in worn teeth treated with direct resin composites. Short‐fiber‐reinforced composites (SFRCs) have been developed as a dentin‐replacing material aiming to improve resistance to crack propagation and increase the toughness of the restored tooth complex. Clinically, similar success rates have been found for direct SFRC and indirect glass ceramic single unit restorations in endodontically treated molars [16]. Traditionally, a layer of conventional resin composite has been recommended to cover SFRC restorations. However, recent in vitro studies demonstrate good wear resistance for SFRC and propose application of the material without proximal coverage [17, 18, 19] and without occlusal coverage [20]. The latter technique has yielded improved fracture behavior [20]. Consequently, it could be presumed that SFRCs could also be used for the management of worn posterior teeth. However, it has yet to be investigated how SFRC would perform when used to cover all cusps, that is, as direct occlusal veneer, and whether there are differences between conventional particulate‐filled resin composites (PFCs) and SFRC when used as occlusal veneers in restoring worn teeth. In addition, SFRC has not yet been used employing the injectable technique. Consequently, the present study was primarily designed to explore the load‐bearing capacity of PFC or SFRC occlusal veneers used as direct restorations in the treatment of worn molars and to analyze the presence of internal voids within the materials. Furthermore, the second aim was to evaluate the effect of water storage on the load‐bearing capacity of the restorations and the correlation between the internal voids and the fracture load. The null hypotheses were that water storage and the presence of internal voids will not affect the load‐bearing capacity of the occlusal veneers fabricated from different direct restorative materials.

## MATERIAL AND METHODS

This study was performed according to CRIS guidelines. Sixty extracted lower human molar teeth were collected and stored in distilled type 1 water until use. According to local regulations and institutional guidelines, ethical permission was not needed for this investigation, as the use of human teeth as samples for medical research does not require an ethical approval when the samples are used without personal data of the donors. Teeth with similar size were selected. The mean dimensions were 11 (±0.5) mm in mesiodistal direction and 9.5 (±0.4) mm in bucco‐oral direction. After removing soft tissue remnants, teeth were embedded in poly(methyl) methacrylate cylinders up to 2 mm below the cementoenamel junction (CEJ). The teeth were randomly divided into six groups (*n* = 10/group) according to restorative material and water storage duration. The sample size was selected based on the literature, without calculating a formal sample size [12, 21, 22]. Teeth in Groups 1 and 2 were restored with flowable PFC (G‐ænial Universal Injectable, GC Corporation) and stored in water for 1 d and 6 months, respectively. Groups 3 and 4 were composed of teeth restored with low‐aspect ratio, that is, micrometer‐scale SFRC (everX Flow Dentin Shade, GC Corporation) and were also stored in water for 1 d and 6 months, respectively. Teeth in Groups 5 and 6 were restored with condensable PFC (Essentia Universal, GC Corporation) and were then stored in water for 1 d and 6 months, respectively.

### Tooth preparation

**FIGURE 1:**
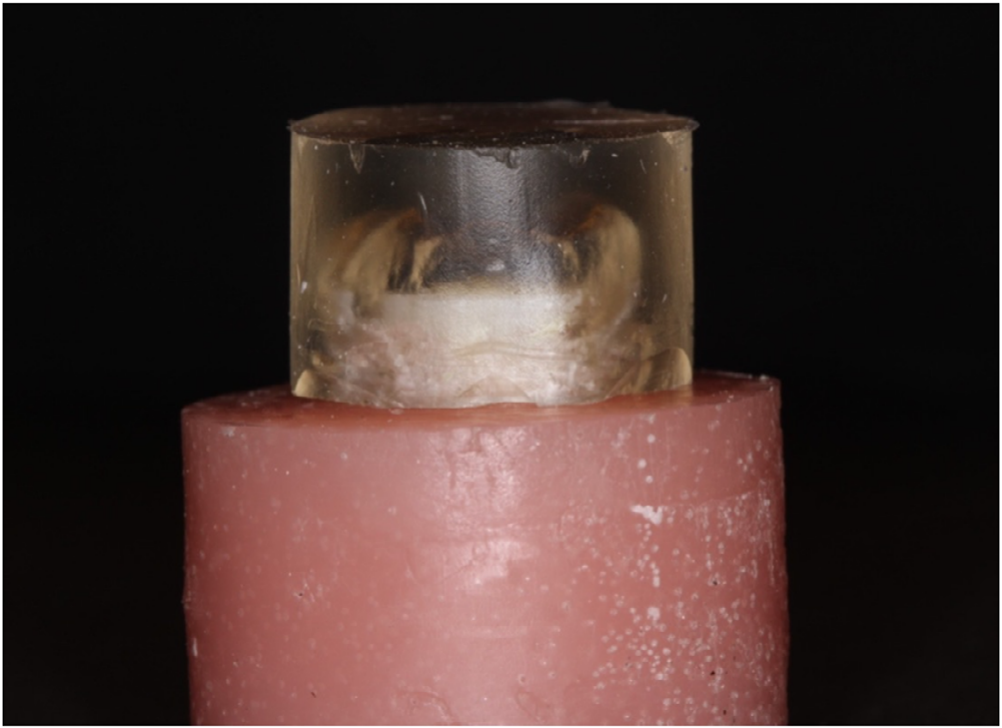
An example of the translucent silicone key used to fabricate the occlusal veneers.

Before tooth preparations, two types of impression keys were obtained from each tooth individually: conventional silicone keys to control the preparation depth and translucent silicone keys to restore the original anatomy of the teeth. Conventional silicone impression keys (Silikon‐Knetmasse, Orbis) were cut in half to serve as preparation indexes, whereas translucent vinyl polysiloxane keys (Exaclear, GC Corporation) were used to fabricate the occlusal veneers (Figure 1). With guidance of the conventional impression keys, flat occlusal surfaces were prepared for each tooth individually until a depth of 2 mm from the central fossa, with only dentin present at the center of the tooth and enamel margins preserved at the outer edges. Preparation type simulated a worn tooth. Preparations were performed under water cooling, using coarse (151‐micron grit) and fine (46‐micron grit) diamond burs (Komet Dental, Gebr. Brasseler GmbH & Co. KG) in a consecutive order. Preparation and restorative procedures were performed by one operator.

### Restorative procedures and group design

The bonding procedure was identical for all teeth. Selective enamel etching was done using 36% phosphoric acid (Blue Etch, Cerkamed) applied for 15 s, then rinsed and gently air‐dried. Primer and adhesive (G2 Bond Universal, GC Corporation) were used as bonding agents. Primer was applied with a microbrush to all the prepared surfaces for 10 s and air‐dried for 5 s, followed by an adhesive layer application. The adhesive layer was applied with a microbrush, then gently air‐dried and light‐cured for 20 s with a light‐curing unit (Elipar LED, TM S10, 3M ESPE) having a light irradiance of 960 mW/cm^2^ and wavelength range of 430–480 nm. The irradiance was measured with a radiometer (Bluephase Meter II, Ivoclar Vivadent) by positioning the tip of the light‐curing unit directly on the center of the sensor. Occlusal veneers were then made from three different resin composites, flowable PFC, condensable PFC, or SFRC, according to the group they belonged to. Materials, used to restore the teeth and their composition, are shown in Table 1.

**TABLE 1:** Materials used in this study and their composition.

Material	Type	Composition	LOT	Manufacturer
Blue Etch	Etchant	36% phosphoric acid	0602231	PPH Cerkamed, Stalowa Wola, Poland
G2‐Bond Universal Primer	Primer	4‐MET, 10‐MDP, DMA, BHT, photoinitiator, acetone, water	2305181	GC Corporation, Tokyo, Japan
G2‐Bond Universal Adhesive	Adhesive	UDMA, DMA, photoinitiator, silica, BHT	2304191	GC Corporation, Tokyo, Japan
G‐ænial Universal Injectable	Flowable nanofilled composite	Resin matrix: dimethacrylate monomers 31 wt% Fillers: barium glass 150 nm and silica (69 wt%)	2203141 2310271 2311061	GC Corporation, Tokyo, Japan
everX Flow dentin shade	Flowable short‐fiber‐reinforced composite	Resin matrix: Bis‐MEPP, TEGDMA, UDMA Fillers: micrometer scale glass fiber (fiber length 140 µm, diameter 6μ m, 25 wt%) and barium glass (42–52 wt%)	2212261 2202021 2310261	GC Corporation, Tokyo, Japan
Essentia Universal	Microfilled hybrid composite	Resin matrix: UDMA, BisEMA, BisGMA, TEGDMA, Bis‐MEPP Fillers: prepolymerized silica (16–17 µm), barium glass and fumed silica (>100 nm, fillers 81 wt%)	2303291 2312051	GC Corporation, Tokyo, Japan

Abbreviations: 10‐MDP, 10‐methacryloyloxydecyl dihydrogen phosphate; 4‐MET, 4‐[2‐(methacryloyloxy)ethoxycarbonyl]phthalic acid; BHT, butylated hydroxytoluene; BisEMA, ethoxylated bisphenol‐A dimethacrylate; BisGMA, bisphenol A‐glycidyl methacrylate; Bis‐MEPP, bisphenol A ethoxylate dimethacrylate; DMA, dimethacrylate; TEGDMA, triethylene glycol dimethacrylate; UDMA, urethane dimethacrylate.

Teeth in Groups 1 and 2 were restored with a flowable PFC (G‐ænial Universal Injectable). First, two perforations through the translucent injection key were made with the tip of the syringe, one for injecting the flowable composite, and another, a relief hole, to allow escape of the excess material and air. The translucent silicone keys used to fabricate the SFRC occlusal veneers in Groups 3 and 4 were prepared in the same way, except that the perforations were made slightly wider using a diamond bur instead of a syringe tip. All teeth in Groups 1–4 were restored using the injectable technique. The translucent impression index was first positioned in place, and the tip of the flowable PFC (G‐ænial Universal Injectable) or SFRC (everX Flow Dentin Shade) syringe was inserted through the created perforation into contact with the tooth and then slightly withdrawn. The flowable resin composite material was slowly injected through the perforation until the excess material began to escape from the relief hole and then light‐polymerized for 20 s on each side through the silicone key. After removing the transparent silicone (injection) key, the excess material was removed with a scalpel and the occlusal veneer light‐polymerized for another 40 s on each side. Teeth in Groups 5 and 6 were restored with condensable PFC (Essentia Universal) using the pressing technique. The translucent silicone key was loaded with a resin composite, positioned on the tooth, pressed, and light‐polymerized through the key for 20 s on each side. After removing the key, the occlusal veneer was further polymerized for 40 s on each side from a distance of approximately 1 mm. These silicone keys were also prepared with two perforations in order to enable the escape of the excess material. Following finishing and polishing procedures with diamond burs and polishing cups, half of the teeth from each material group were stored in distilled water at 37°C for 1 d (Groups 1, 3, and 5) and the other half for 6 months (Groups 2, 4, and 6) prior to loading test.

### Loading test

The quasi‐static loading test was performed in air at room temperature using an LR30K Plus loading machine with a 30‐kN loadcell (Lloyd Instruments/Ametek). Each restored tooth was loaded through a 5.5 mm diameter steel ball along the long axis of the tooth with a crosshead speed of 1 mm/min until fracture. Fracture load values were measured in newtons (N). The load‐deformation curves were recorded with the computer software connected to the testing machine and utilized to analyze the fracture behavior of the materials. Fracture types were visually analyzed by two operators and classified into two fracture modes: (1) repairable, which included cohesive fracture of the restorative material with either superficial fracture (delamination) of the enamel or intact tooth structure and cohesive fracture of the restored tooth above or at the CEJ and (2) non‐repairable (catastrophic), which included cohesive fracture of the tooth below the CEJ and root fracture.

### Micro‐computed tomography (µCT) analysis

For determining possible internal voids within each occlusal veneer, teeth (*n* = 10/material) that were stored in water for 6 months were imaged using micro‐computed tomography (µCT) analysis (Bruker Skyscan 1272). Each sample was wrapped in parafilm sealing film to limit water evaporation during scanning. Scanning parameters for restored teeth were set at 80 kV source voltage, 125 µA source current, 1 mm aluminum filter, 9.0 µm pixel size, and 0.200° rotation step. Cross‐sectional images were reconstructed using nrecon 1.7.5.6 software with the following settings: attenuation coefficient range 0.005–0.100, smoothing four, ring artifact reduction six, and beam hardening reduction 60%. The analysis of internal void volumes within the material was performed by ctan 1.23.0.2 software (Bruker SkyScan 1272). The voids were identified by selecting threshold values 0–10 of the 8‐bit grayscale images. Visualization of scanned teeth and voids within the material was performed with CTVox 3.3.1 image rendering program (Bruker Skyscan 1272).

### Statistical analysis

Shapiro–Wilk test was used to assess the normality of the fracture load data distribution according to material and water storage duration. The fracture load data were statistically analyzed using two‐way analysis of variance followed by multiple comparisons by the Tukey HSD test. Welch's test followed by the Tukey HSD test was used to compare internal voids‐to‐material volume ratio between materials. Internal void analyses were performed using JMP 17 Pro program (JMP, Version 17. SAS Institute, 1989–2023). Correlation between fracture load and internal voids‐to‐material volume ratio was evaluated using Pearson correlation (IBM SPSS Statistics 29). A *p‐*value less than 0.05 was considered statistically significant.

## RESULTS

**FIGURE 2:**
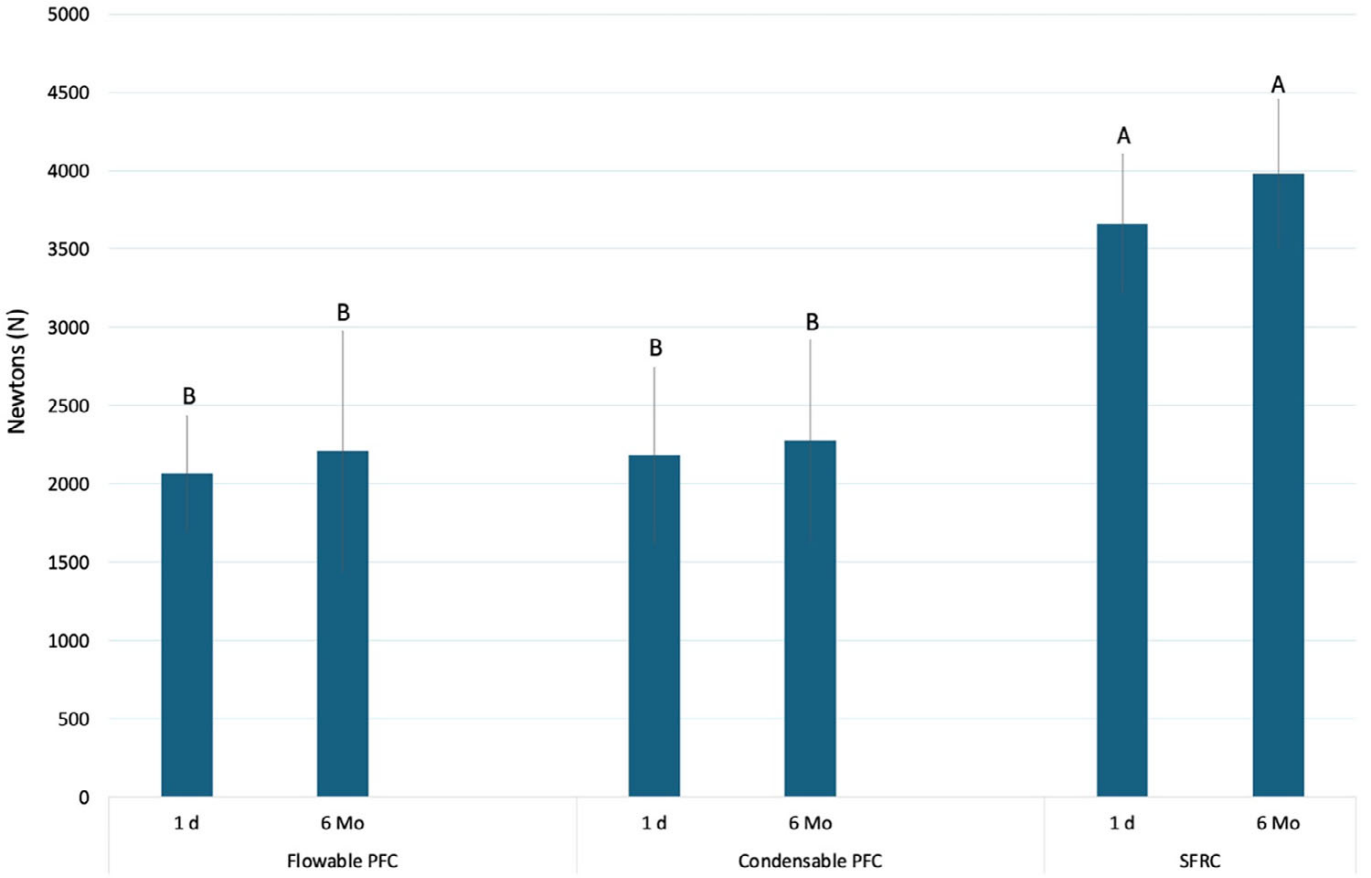
Fracture load values of tested restorations. Different letters (A and B) above the columns indicate statistically significant difference. Teeth were stored 1 d and 6 months in water at 37°. Flowable PFC: G‐ænial Universal Injectable; Condensable PFC: Essentia Universal; SFRC: everX Flow. PFC, particulate filler composite; SFRC, short‐fiber‐reinforced composite.

**FIGURE 3:**
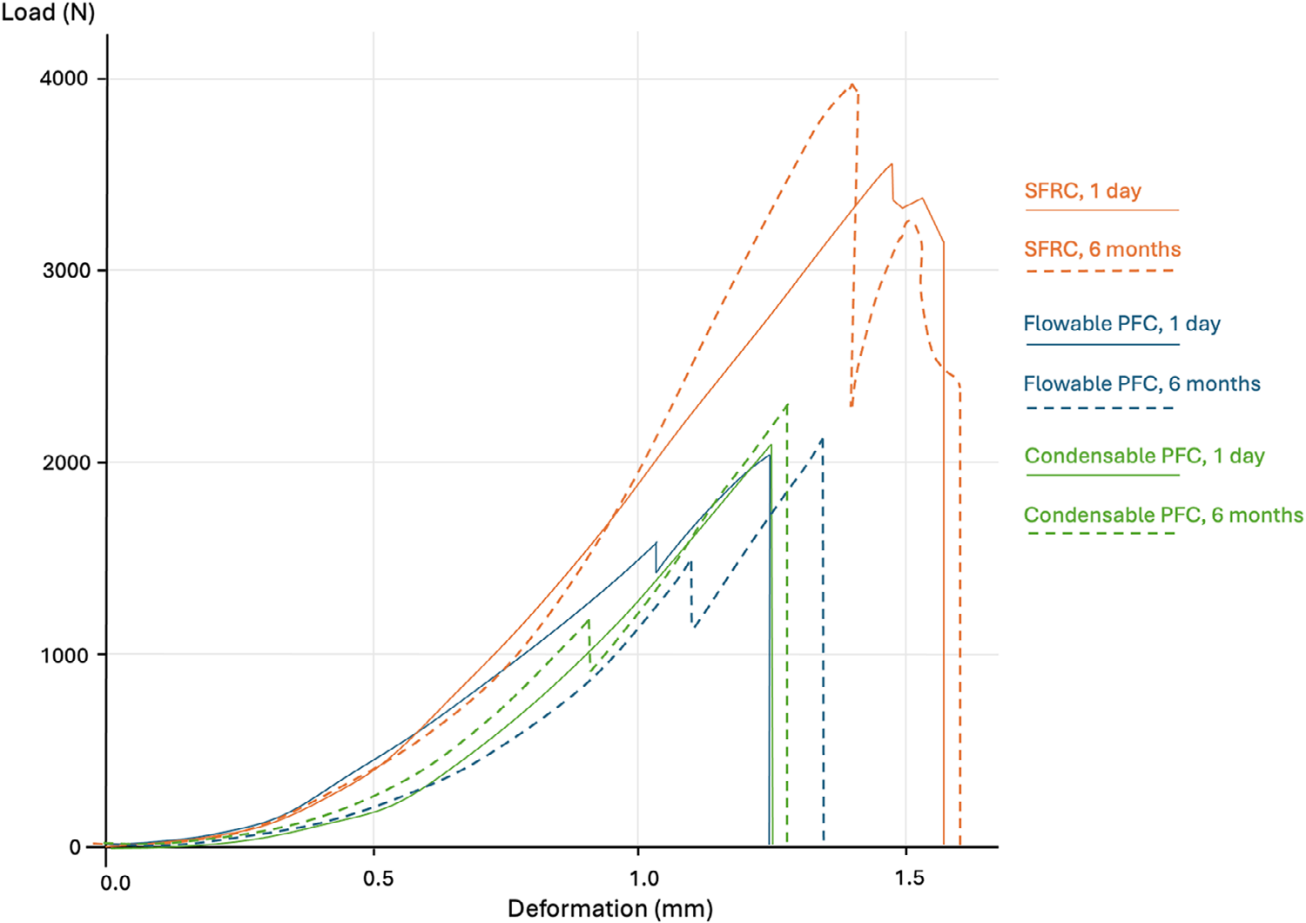
Load‐deformation curves for tested restorations. Teeth were stored 1 d and 6 months in water at 37°. SFRC: everX Flow; Flowable PFC: G‐ænial Universal Injectable; Condensable PFC: Essentia Universal. PFC, particulate filler composite; SFRC, short‐fiber‐reinforced composite.

The results of the static loading test are summarized in Figure 2. SFRC occlusal veneers exhibited the highest fracture load values irrespective of the duration time in water (*p* < 0.0001). Fracture load values after 1 d of water storage were 3660 ± 446 N for SFRC (everX Flow), 2183 ± 563 N for the condensable PFC (Essentia Universal), and 2064 ± 372 N for the flowable PFC (G‐ænial Universal). Respectively, the load values after 6 months of water storage were 3979 ± 476 N for SFRC, 2274 ± 647 N for Essentia Universal, and 2206 ± 372 N for G‐ænial Universal. Water storage duration had no significant influence on fracture load (*p* > 0.05). However, in all groups, prolonged water storage increased the fracture load (Figure 2). According to load‐deformation curves, teeth restored with SFRC occlusal veneers failed after reaching the first peak of the load curve, which was also the highest peak. In contrast, teeth restored with PFC occlusal veneers failed instantly at the highest peak, which was not the first peak in the load curve (Figure 3).

All restored teeth fractured predominantly catastrophically (Table 2). SFRC occlusal veneers presented 10%–40% less catastrophic fractures than teeth restored with PFC occlusal veneers (Table 2). Visually analyzed, fractures in all cases were located on the occlusal surface at the contact area of the loading ball.

**TABLE 2:** Fracture type analysis with the failure mode presented in percent (%).

	Repairable		Non‐repairable
	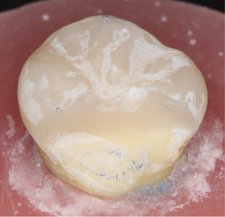	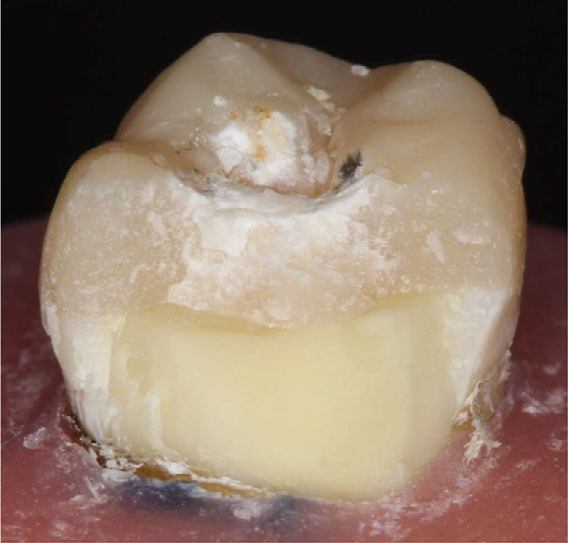	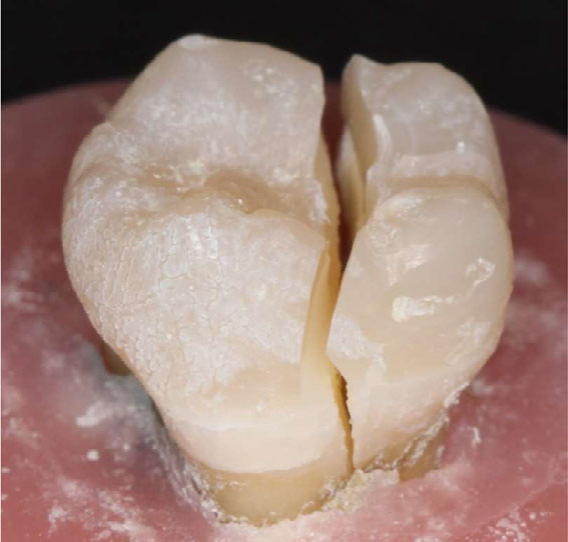
**Flowable PFC**			
1 d 6 months	30% 40%		70% 60%
**SFRC**			
1 d 6 months	60% 40%		40% 60%
**Condensable PFC**			
1 d 6 months	20% 30%		70% 80%

*Note*: Teeth were stored 1 d and 6 months in water at 37°.

Abbreviations: PFC, particulate filler composite; SFRC, short‐fiber‐reinforced composite.

**FIGURE 4:**
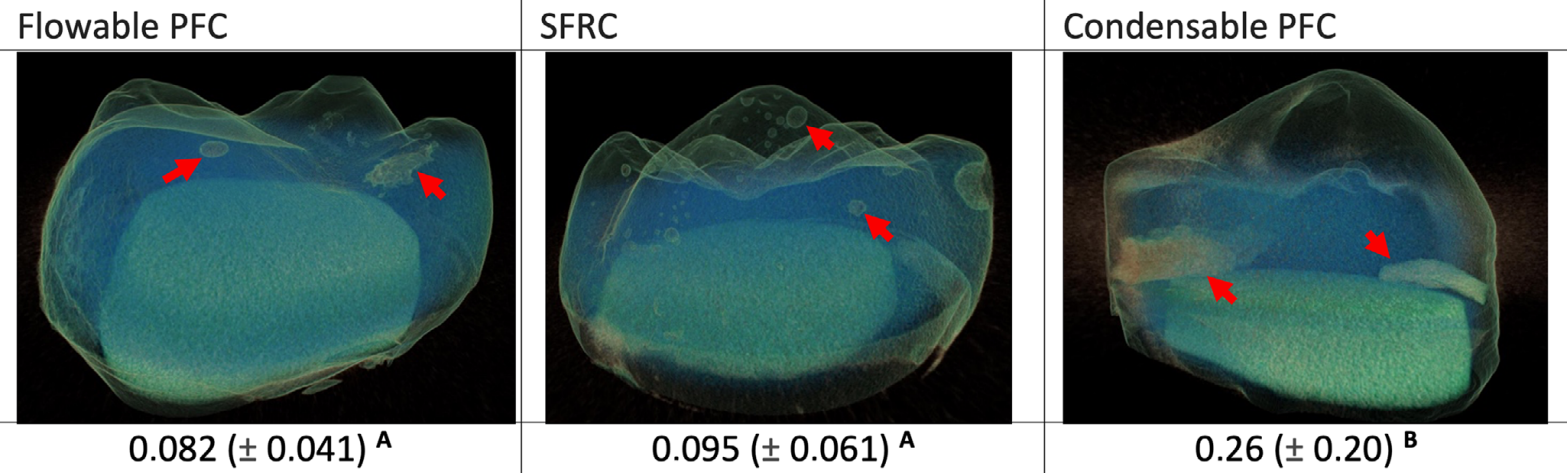
Occlusal veneers scanned with micro‐computed tomography (µCT) revealed internal voids within the material, indicated by red arrows. After each µCT image presented is the corresponding value of the internal voids‐to‐restorative material volumetric ratio and standard deviations (SDs). Different superscript letters indicate statistically significant difference. Flowable PFC: G‐ænial Universal Injectable; SFRC: everX Flow; Condensable PFC: Essentia Universal. PFC, particulate filler composite; SFRC, short‐fiber‐reinforced composite.

**FIGURE 5:**
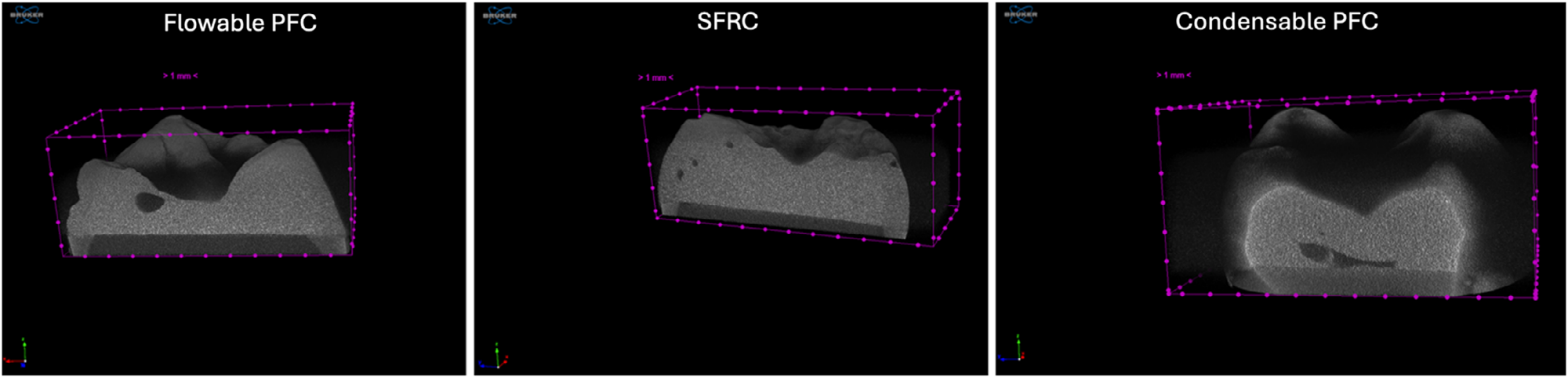
Cross‐sectional view of the occlusal veneers scanned with micro‐computed tomography (µCT), which revealed internal voids within the material. Flowable PFC: G‐ænial Universal Injectable; SFRC: everX Flow; Condensable PFC: Essentia Universal. PFC, particulate filler composite; SFRC, short‐fiber‐reinforced composite.

The µCT analysis of the internal voids showed material‐type‐dependent characteristics (Figures 4 and 5). In both flowable composites, small spherical internal voids (i.e., air bubbles) were located superficially, at the outer layers of the occlusal veneers, whereas in condensable PFC, larger and irregular voids were found at the bottom layer of the occlusal veneers, that is, the restoration—tooth interface (Figures 4 and 5). In terms of internal voids‐to‐material volume ratio, occlusal veneers made from condensable PFC presented a significantly higher volumetric ratio (*p* ≤ 0.027) than flowable PFC or SFRC occlusal veneers (Figure 4). Although correlations between the fracture load values and the internal voids‐to‐material volume ratio were not significant, a slight negative correlation was found. Recorded values were −0.065 (*p* = 0.859), −0.091 (*p* = 0.803), and −0.199 (*p* = 0.582) for flowable PFC, SFRC, and condensable PFC, respectively. This outcome indicates that single internal voids within the material might not significantly affect the fracture load.

## DISCUSSION

Rehabilitation of worn dentition has traditionally involved direct resin composite restorations only as interim restorations during the tentative period of the increased vertical dimension. Direct restorations have been considered medium‐term provisional option as they are reversible and easy to adjust but too vulnerable to technical complications such as chipping, fracture, wear, and debonding to be retained permanently [23]. However, as dental resin composite materials have evolved in terms of filler technology and continuous tailoring of the monomer and initiator systems, these materials have become an acceptable material choice to treat posterior tooth wear [24, 25]. Subsequently, in the present study, three different direct resin composite materials were used as definitive restorative option for restoring worn posterior teeth (molars). Both null hypotheses were accepted. Prolonged water storage (6 months) and the presence of internal voids did not affect the load‐bearing capacity of any of the direct restorative materials investigated in this study. The only variable that affected the load‐bearing capacity was the material type. Teeth restored with SFRC occlusal veneers exhibited statistically significantly higher fracture load compared with both PFCs, regardless of the duration of storage time in water. Furthermore, higher relation of internal voids‐to‐restorative material was observed for the condensable PFC compared with both flowable materials.

The flowable PFC (G‐ænial Universal Injectable) is a nanofilled composite; the condensable PFCs (Essentia Universal) are microhybrid composite with pre‐polymerized micro‐sized fillers; and the flowable SFRC (everX Flow) is a randomly oriented short‐fiber composite with micrometer‐scale short E‐glass fibers (length 140 µm, diameter 6 µm, aspect ratio 23) and micro‐sized particulate fillers (Table 1). Different filler size and distribution [11], inter‐filler distance [26], filler‐resin interfacial adhesion [27], silane type, and monomer used [28] affect various mechanical properties that are used to predict the clinical failures of a material, such as wear and fractures [29, 30]. For example, in order to withstand the occlusal load without bending, deformation, or debonding, the flexural strength of a restorative material intended to be used in stress‐bearing areas must exceed 80 MPa [31]. Corresponding required values for flexural modulus, which describes the resistance to deformation under load, and fracture toughness, which describes the resistance to crack propagation, are 5–10 GPa and 0.5–0.9 MPa m^1/2^, respectively [32]. The materials used in this study have values that are within the range or exceed the reference values for the properties mentioned above. Namely, the flowable nanofilled composite used in this study, G‐ænial Universal Injectable, and the microhybrid composite, Essentia Universal, have almost equivalent values for flexural strength ca. 120 MPa, but their flexural moduli are 7.2 and 12.9 GPa, respectively [33]. The flowable short‐fiber composite (everX Flow) has a flexural strength of 146.5 MPa and a flexural modulus of 9 GPa [33]. Moreover, the fracture toughness values for G‐ænial Universal Injectable, Essentia Universal, and everX Flow are 1, 1.1, and 2.8 MPa m^1/2^, respectively [33].

Despite the significantly lower flexural modulus of the flowable nanofilled composite (G‐ænial Universal Injectable), differences in the fracture load or the fracture type between the two particulate filler composites were not observed. This could be due to the sufficient thickness in which the composite material was applied, which most likely contributed to resisting flexural deformation. Otherwise, if applied in a thin layer, a composite with low flexural modulus will clinically result in fracture (either loss of the restoration or catastrophic failure), as such a material will deform easily under load.

SFRC occlusal veneers exhibited significantly higher fracture load and less catastrophic fractures compared to both PFC occlusal veneers. A group of correlated factors rather than one factor could explain the results. These factors are the reinforcing nature of short‐fiber fillers, known for carrying the load transferred from the resin matrix to the fibers (when properly bonded to resin matrix through silane), their aspect ratio, fiber loading, and fiber orientation [34]; and also the sufficient thickness of the SFRC layer and the close vicinity of the short fibers to the surface where the load was applied. As a result, the short‐fiber fillers with random alignment could redirect the crack coming from the occlusal surface, a process that relieves the stress concentrated at the crack tip and dissipates energy by fiber pullout. In fact, typical fracture mechanism for low aspect ratio SFRC is fiber pull‐out during crack opening and matrix cracking [35]. As this process consumes energy, retardation of a crack could take place, and, at the breaking point, the fracture might be arrested by fiber that crosses or interferes with its direction [35]. These events constitute the characteristic step‐wise load‐deformation curve for SFRCs [35]. In the present study, however, the typical step‐wise curve was not observed, probably due to the different specimen geometry. The tail, detected after cracking has taken place, is only a narrow region affected most likely by the fiber orientation distribution and is not a real plastic zone (Figure 3). The crack in PFC occlusal veneers propagated and caused matrix fractures in the resin matrix, which are denoted as initial peaks in the curves, and then continued between and circumvented the filler particles. Nevertheless, when comparing the load‐deflection curves of SFRC and both PFCs, it is apparent that the former demonstrated a significant rise in the load‐bearing capacity before fracturing. This is supported by the fracture type analysis, which showed that teeth restored with SFRC occlusal veneers also fractured in a slightly less destructive pattern (10%–40% less catastrophic fractures) and that short‐fiber fillers directed fractures towards the periphery of the restoration. This finding is in line with earlier studies [36, 37].

Water uptake in resin composites is known to decrease their mechanical properties, primarily due to the degradation of chemical bonds at the resin‐filler interface and the plasticization of the polymer matrix [38]. Indeed, hydrolytic degradation between resin monomers in resin matrix and between resin monomer‐filler interface decreases elastic modulus and initial fracture strength of resin composite [39]. However, FRCs appear to be less affected by water exposure compared to PFCs, likely due to the stable matrix network and/or strong fiber‐matrix adhesion [40]. The deleterious effect of prolonged storage in water was not observed in the present study for any material. In fact, 6 months of water storage slightly increased fracture load values, which could be partly explained by the post‐curing of resin composite or by the fact that water sorption plasticized the polymer matrix, which increased resistance against fracture under loading. Post‐curing refers to the continuation of polymerization after the initial light‐curing. Following light exposure, resin composites continue to react as the degree of conversion of residual monomers increases over time through ongoing densification and polymer chain cross‐linking. This leads to a higher degree of cure and improved hardness, mechanical strength, and fracture resistance compared with immediately light‐polymerized specimens [41, 42]. However, differences between fracture load values between 1 d and 6 months of water storage groups for each material individually were not statistically significant. Alrahlah *et al.* [43] showed that hygroscopic expansion in resin composites, which reflects water absorption, reached equilibrium after 60 d [43]. This supports the use of a 6‐month water storage period in the current study. Although water storage alone cannot fully reproduce the complex aging processes in vivo, it provides a controlled method to assess the potential effects of moisture on material stability and polymer degradation.

Inherent flaws such as voids with entrapped oxygen may serve as sites for crack initiation. Porosity is linked to restoration weakening and material degradation [44, 45]. Hence, µCT analysis was conducted to assess the internal defects of the direct occlusal veneers. It was interesting to observe that the internal voids in both flowable composites were small and spherical and located superficially or at the subsurface. On the other hand, larger and irregular voids were found at the bottom layer of the occlusal veneers made from condensable composite (Figures 4 and 5). The restorative technique itself was most likely an important factor affecting the void formation at these places. The resin composites were placed in one single increment. Flowable composites were injected, and voids probably developed due to nonhomogeneous flow and air trapping when removing the injection tip. The pressing (seating) technique was used to apply the condensable composite. In this technique, the composite was placed in the index and the index was placed on the tooth and then seated with finger pressure. The void's formation at the bottom layer could result from misfitted of misfiled matrix, compromising the integrity of the restoration—tooth interface. Possibly placing the composite on the tooth and then pressing the index on the tooth could have resulted in different outcomes. Fractures originating from the subsurface could be repairable as they are located at the outer layer, whereas a defect at the bottom layer would most likely lead to debonding, chipping, or cohesive material fracture that in most cases would lead to restoration replacement.

These voids did not significantly affect the load‐bearing capacity, however. Correlation analysis between the fracture load values and the internal voids‐to‐restorative material volumetric ratio showed a slightly negative correlation, suggesting that small and single voids (individual air bubbles) do not yet impair the load‐bearing capacity. However, one might assume that an increased amount of air, for example, coalesced voids into a cavity, could reduce the fracture load. Only limited research is available on the effect of internal voids on the marginal adaptation and interfacial gap formation [46]. Hence, future research could focus on the internal voids effect on the fracture endurance and wear of direct resin composite restorations under different aging and chewing conditions.

This study was performed according to CRIS guidelines to promote quality and transparency of the study. However, the results of the present study need cautious interpretation and should be analyzed within the scope of the study. Limitations of this in vitro research are the absence of sample size calculation, thermal aging, and chewing or dynamic conditions. The number of specimens per group was decided based on the literature, which has shown that this size allows detection of statistically significant difference because of the homogeneity of the in vitro testing conditions. The static load test employed in this study is a destructive test, which does not represent the typical forces in the oral cavity and leads to catastrophic failures. Nonetheless, linear relationship between static and fatigue loading has been shown to exist [47, 48]. The present study, however, utilized this test to understand how the composition of the material and the quality of the restoration (internal voids) affect the fracture behavior. Indeed, static tests are also undertaken to understand the relationship between the strength and the microstructural features of the material. In addition, the load‐deformation curves were interpreted with great care in the present investigation. Hence, data could be used to understand the relationship between the composition and consistency of the material and the restorative technique. Future studies are needed to evaluate the functional aspects of these types of restorations under more complex loading conditions. Another limitation of the present study is that a scanning electron microscopy fractography analysis is missing. This analysis would have enabled detection of fracture features such as fracture origin, thickness, and propagation pattern. Additional limitation is that a group with a bi‐layered SFRC structure is missing. In this design, the flowable SFRC would have been veneered with a layer of conventional resin composite, as recommended by the manufacturer. Furthermore, simulating a periodontal ligament could have allowed a natural movement of a tooth under stress. It is also noteworthy that light‐curing distance through the translucent silicone impression material varied; however, the occlusal veneers were additionally light‐polymerized from each side after removing the translucent silicone matrix.

Within the limitations of this study, it can be concluded that use of SFRC resin as occlusal veneers for restoring worn molars can significantly enhance their load‐bearing capacity. Prolonged water storage did not significantly affect the load‐bearing capacity, and single internal voids did not significantly impact the fracture load. Short‐fiber fillers redirected fractures towards the periphery of the restoration.

## AUTHOR CONTRIBUTIONS

Pekka K. Vallittu, Timo Närhi, Anna‐Maria Le Bell‐Rönnlöf, Jasmina Bijelic‐Donova, and Topias Yli‐Urpo contributed to the study conception and design. Topias Yli‐Urpo and Jasmina Bijelic‐Donova performed laboratory work and data collection. Jorma Määttä performed micro‐CT analysis. Auli Suominen and Topias Yli‐Urpo performed statistical analysis. The first draft of the manuscript was written by Topias Yli‐Urpo, which was reviewed and edited by Jasmina Bijelic‐Donova, Anna‐Maria Le Bell‐Rönnlöf, Pekka K. Vallittu, and Timo Närhi. All authors have read and approved the final manuscript.

## CONFLICT OF INTEREST STATEMENT

P.V. declares that he consults Stick Tech—Member of GC in training and RD. All authors declare no conflicts of interest.

## References

[1] Warreth A , Abuhijleh E , Almaghribi MA , Mahwal G , Ashawish A . Tooth surface loss: a review of literature. Saudi Dent J. 2020;32:53–60. 10.1016/j.sdentj.2019.09.004 PMC7016226 32071532

[2] Salari N , Sadeghi AH , Abdolmaleki A , Zarei H , Ghaderi AH , Shohaimi S , et al. The global prevalence of tooth wear in general population: a systematic review and meta‐analysis. Public Health Pract (Oxf) 2025;11:100708. 10.1016/j.puhip.2025.100708 PMC12771503 41502587

[3] Loomans B , Opdam N , Attin T , Bartlett D , Edelhoff D , Frankenberger R , et al. Severe tooth wear: European consensus statement on management guidelines. J Adhes Dent. 2017;19:111–9. 10.3290/j.jad.a38102 28439579

[4] Mehta SB , Banerji S , Millar BJ , Suarez‐Feito JM . Current concepts on the management of tooth wear: part 4. An overview of the restorative techniques and dental materials commonly applied for the management of tooth wear. Br Dent J. 2012;212:169–77. 10.1038/sj.bdj.2012.137 22361546

[5] Varma S , Preiskel A , Bartlett D . The management of tooth wear with crowns and indirect restorations. Br Dent J. 2018;224:343–7. 10.1038/sj.bdj.2018.170 29495030

[6] Josic U , D'Alessandro C , Miletic V , Maravic T , Mazzitelli C , Jacimovic J , et al. Clinical longevity of direct and indirect posterior resin composite restorations: an updated systematic review and meta‐analysis. Dent Mater. 2023;39:1085–94. 10.1016/j.dental.2023.10.009 37827872

[7] Hofsteenge JW , Fennis WMM , Kuijs RH , Özcan M , Cune MS , Gresnigt MMM , et al. Clinical survival and performance of premolars restored with direct or indirect cusp‐replacing resin composite restorations with a mean follow‐up of 14 years. Dent Mater. 2023;39:383–90. 10.1016/j.dental.2023.03.004 36959076

[8] Mehta SB , Banerji S , Millar BJ , Suarez‐Feito JM . Current concepts on the management of tooth wear: part 4. An overview of the restorative techniques and dental materials commonly applied for the management of tooth wear. Br Dent J. 2012;212:169–77. 10.1038/sj.bdj.2012.137 22361546

[9] Alzraikat H , Burrow M , Maghaireh G , Taha N . Nanofilled resin composite properties and clinical performance: a review. Oper Dent. 2018;43:E173–E90. 10.2341/17-208-T 29570020

[10] Kim KH , Ong JL , Okuno O . The effect of filler loading and morphology on the mechanical properties of contemporary composites. J Prosthet Dent. 2002;87:642–9. 10.1067/mpr.2002.125179 12131887

[11] Randolph LD , Palin WM , Leloup G , Leprince JG . Filler characteristics of modern dental resin composites and their influence on physico‐mechanical properties. Dent Mater. 2016;32:1586–99. 10.1016/j.dental.2016.09.034 27720423

[12] Tribst JPM , Tach Q , De Kok P , Dal Piva AMDO , Kuijs RH , Kleverlaan CJ . Thickness and substrate effect on the mechanical behaviour of direct occlusal veneers. Int Dent J. 2023;73:612–9. 10.1016/j.identj.2022.11.006 PMC10509395 36509557

[13] Hardan L , Mancino D , Bourgi R , Cuevas‐Suárez CE , Lukomska‐Szymanska M , Zarow M , et al. Treatment of tooth wear using direct or indirect restorations: a systematic review of clinical studies. Bioengineering (Basel) 2022;9:346. 10.3390/bioengineering9080346 PMC9404995 36004871

[14] Bartlett D , Varma S . A retrospective audit of the outcome of composites used to restore worn teeth. Br Dent J. 2017;223:33–6. 10.1038/sj.bdj.2017.583 28684832

[15] Tauböck TT , Schmidlin PR , Attin T . Vertical bite rehabilitation of severely worn dentitions with direct composite restorations: clinical performance up to 11 years. J Clin Med. 2021;10:1732. 10.3390/jcm10081732 PMC8073648 33923679

[16] Bijelic‐Donova J , Myyryläinen T , Karsila V , Vallittu PK , Tanner J . Direct short‐fiber reinforced composite resin restorations and glass‐ceramic endocrowns in endodontically treated molars: a 4‐year clinical study. Eur J Prosthodont Restor Dent. 2022;30:284–95. 10.1922/EJPRD_2333Bijelic-Donova12 35438265

[17] ElAziz RHA , ElAziz SAA , ElAziz PMA , Frater M , Vallittu PK , Lassila L , et al. Clinical evaluation of posterior flowable short fiber‐reinforced composite restorations without proximal surface coverage. Odontology 2024;112:1274–83. 10.1007/s10266-024-00905-5 PMC11415407 38393515

[18] Lassila L , Säilynoja E , Prinssi R , Vallittu P , Garoushi S . Characterization of a new fiber‐reinforced flowable composite. Odontology 2019;107:342–52. 10.1007/s10266-018-0405-y PMC6557871 30617664

[19] Lassila L , Novotny R , Säilynoja E , Vallittu PK , Garoushi S . Wear behavior at margins of direct composite with CAD/CAM composite and enamel. Clin Oral Investig. 2023;27:2419–26. 10.1007/s00784-023-04883-w PMC10159970 36746817

[20] Jakab A , Palkovics D , Szabó VT , Szabó B , Vincze‐Bandi E , Braunitzer G , et al. Mechanical performance of extensive restorations made with short fiber‐reinforced composites without coverage: a systematic review of in vitro studies. Polymers (Basel) 2024;16:590. 10.3390/polym16050590 PMC10934356 38475274

[21] Aboelnor MM , Nour KA , Al‐Sanafawy HMA . Fracture strength of direct occlusal veneers with different short fiber‐reinforced composite cores and veneering materials: an in‐vitro study. Clin Oral Investig. 2024;28:635. 10.1007/s00784-024-06013-6 PMC11551077 39523240

[22] Rocca GT , Saratti CM , Cattani‐Lorente M , Feilzer AJ , Scherrer S , Krejci I . The effect of a fiber reinforced cavity configuration on load bearing capacity and failure mode of endodontically treated molars restored with CAD/CAM resin composite overlay restorations. J Dent. 2015;43:1106–15. 10.1016/j.jdent.2015.06.012 26149065

[23] Bartlett D , Sundaram G . An up to 3‐year randomized clinical study comparing indirect and direct resin composites used to restore worn posterior teeth. Int J Prosthodont. 2006;19:613–7. 17165303

[24] Hamburger JT , Opdam NJ , Bronkhorst EM , Kreulen CM , Roeters JJ , Huysmans MC . Clinical performance of direct composite restorations for treatment of severe tooth wear. J Adhes Dent. 2011;13:585–93. 10.3290/j.jad.a22094 21935514

[25] Loomans BAC , Kreulen CM , Huijs‐Visser HECE , Sterenborg BAMM , Bronkhorst EM , Huysmans MCDNJM , et al. Clinical performance of full rehabilitations with direct composite in severe tooth wear patients: 3.5 years results. J Dent. 2018;70:97–103. 10.1016/j.jdent.2018.01.001 29339203

[26] Shen J , Lin X , Liu J , Li X . Revisiting stress–strain behavior and mechanical reinforcement of polymer nanocomposites from molecular dynamics simulations. Phys Chem Chem Phys. 2020;22:16760–71. 10.1039/d0cp02225j 32662467

[27] Rodríguez HA , Kriven WM , Casanova H . Development of mechanical properties in dental resin composite: effect of filler size and filler aggregation state. Mater Sci Eng C Mater Biol Appl. 2019;101:274–82. 10.1016/j.msec.2019.03.090 31029321

[28] Pratap B , Gupta RK , Bhardwaj B , Nag M . Resin based restorative dental materials: characteristics and future perspectives. Jpn Dent Sci Rev. 2019;55:126–38. 10.1016/j.jdsr.2019.09.004 PMC6819877 31687052

[29] Ferracane JL . Resin‐based composite performance: are there some things we can't predict? Dent Mater. 2013;29:51–8. 10.1016/j.dental.2012.06.013 PMC3495244 22809582

[30] Heintze SD , Ilie N , Hickel R , Reis A , Loguercio A , Rousson V . Laboratory mechanical parameters of composite resins and their relation to fractures and wear in clinical trials—a systematic review. Dent Mater. 2017;33:e101–e14. 10.1016/j.dental.2016.11.013 27993372

[31] ISO 4049:2019 , Dentistry—polymer‐based restorative materials . Geneva: International Organization for Standardization; 2019.

[32] Abdulhameed N , Angus B , Wanamaker J , Mecholsky JJ . Quantitative fractography as a novel approach to measure fracture toughness of direct resin composites. J Mech Behav Biomed Mater. 2020;109:103857. 10.1016/j.jmbbm.2020.103857 32543417

[33] Lassila L , Säilynoja E , Prinssi R , Vallittu PK , Garoushi S . Fracture behavior of Bi‐structure fiber‐reinforced composite restorations. J Mech Behav Biomed Mater. 2020;101:103444. 10.1016/j.jmbbm.2019.103444 31561057

[34] Khan AA , Zafar MS , Fareed MA , AlMufareh NA , Alshehri F , AlSunbul H , et al. Fiber‐reinforced composites in dentistry—an insight into adhesion aspects of the material and the restored tooth construct. Dent Mater. 2023;39:141–51. 10.1016/j.dental.2022.12.003 36604257

[35] Bijelic‐Donova J , Garoushi S , Lassila LV , Rocca GT , Vallittu PK . Crack propagation and toughening mechanism of bilayered short‐fiber reinforced resin composite structure—evaluation up to six months storage in water. Dent Mater J. 2022;41:580–8. 10.4012/dmj.2021-321 35584936

[36] Garoushi S , Mangoush E , Vallittu M , Lassila L . Short fiber reinforced composite: a new alternative for direct onlay restorations. Open Dent J. 2013;7:181–5. 10.2174/1874210601307010181 PMC3915317 24511331

[37] Garoushi S , Akbaşak‐Sungur AÖ , Erkut S , Vallittu PK , Uctasli S , Lassila L . Evaluation of fracture behavior in short fiber–reinforced direct and indirect overlay restorations. Clin Oral Investig. 2023;27:5449–58. 10.1007/s00784-023-05164-2 PMC10492695 37477724

[38] Curtis AR , Shortall AC , Marquis PM , Palin WM . Water uptake and strength characteristics of a nanofilled resin‐based composite. J Dent. 2008;36:186–93. 10.1016/j.jdent.2007.11.015 18237839

[39] Lohbauer U , Frankenberger R , Krämer N , Petschelt A . Time‐dependent strength and fatigue resistance of dental direct restorative materials. J Mater Sci Mater Med. 2003;14:1047–53. 10.1023/b:jmsm.0000004001.73640.4c 15348497

[40] Tiu J , Belli R , Lohbauer U . R‐curve behavior of a short‐fiber reinforced resin composite after water storage. J Mech Behav Biomed Mater. 2016;104:103674. 10.1016/j.jmbbm.2020.103674 32174430

[41] Cook WD , Johannson M . The influence of postcuring on the fracture properties of photo‐cured dimethacrylate based dental composite resin. J Biomed Mater Res. 1987;21:979–89. 10.1002/jbm.820210804 3654690

[42] Carek A , Dukaric K , Miler H , Marovic D , Tarle Z , Par M . Post‐cure development of the degree of conversion and mechanical properties of dual‐curing resin cements. Polymers 2022;14:3649. 10.3390/polym14173649 PMC9460751 36080725

[43] Alrahlah A , Silikas N , Watts DC . Hygroscopic expansion kinetics of dental resin‐composites. Dent Mater. 2014;30:143–8. 10.1016/j.dental.2013.10.010 24268572

[44] Balthazard R , Jager S , Dahoun A , Gerdolle D , Engels‐Deutsch M , Mortier E . High‐resolution tomography study of the porosity of three restorative resin composites. Clin Oral Investig. 2014;18:1613–8. 10.1007/s00784-013-1139-4 24287890

[45] Opdam N . Porosities and voids in Class I restorations placed by six operators using a condensable or syringable composite. Dent Mater. 2002;18:58–63. 10.1016/s0109-5641(01)00020-3 11740965

[46] Baldi A , Rossi T , Comba A , Monticone L , Paolone G , Sannino I , et al. Three‐dimensional internal voids and marginal adaptation in deep margin elevation technique: efficiency of highly filled flowable composites. J Adhes Dent. 2024;26:223–30. 10.3290/j.jad.b5759489 PMC11748039 39397757

[47] Bijelic‐Donova J , Garoushi S , Vallittu PK , Lassila LV . Mechanical properties, fracture resistance, and fatigue limits of short fiber reinforced dental composite resin. J Prosthet Dent. 2016;115:95–102. 10.1016/j.prosdent.2015.07.012 26460170

[48] Garoushi S , Lassila LV , Tezvergil A , Vallittu PK . Static and fatigue compression test for particulate filler composite resin with fiber‐reinforced composite substructure. Dent Mater. 2007;23:17–23. 10.1016/j.dental.2005.11.041 16414110

